# Prevalence of thyroid nodules in an occupationally radiation exposed group: a cross sectional study in an area with mild iodine deficiency

**DOI:** 10.1186/1471-2458-5-73

**Published:** 2005-07-07

**Authors:** Paolo Trerotoli, Anna Ciampolillo, Giuseppe Marinelli, Riccardo Giorgino, Gabriella Serio

**Affiliations:** 1 Department of Internal Medicine and Public Medicine, Chair of Medical Statistic, University of Bari, Italy; 2 Department of Emergency and Transplantation, Chair of Endocrinology, University of Bari, Italy; 3 Radioprotection Staff, Direzione Sanitaria, Azienda Ospedaliera Policlinico, Bari, Italy

## Abstract

**Background:**

Thyroid nodules and thyroid cancer occur more frequently in people exposed to radiation for therapeutic purposes, and to nuclear fallout. Furthermore, it is known that a moderate degree of iodine deficiency may be responsible for an increased prevalence of thyroid nodules, while it is suspected that radiation exposure could induce changes in thyroid autoimmunity. The iodine intake of people resident in Bari, S. Italy, is mildly deficient, which could be presumed to cause a higher prevalence of thyroid pathology. This study was conducted to evaluate the prevalence of thyroid nodules in a population occupationally exposed to radiation, in an area of mild iodine deficiency.

**Methods:**

A cross-sectional study was designed to evaluate the prevalence of thyroid nodules in radiation exposed workers, compared with a stratified sample of non exposed workers. After giving written consent to participate in the study, all the recruited subjects (304 exposed and 419 non exposed volunteers) were interviewed to fill in an anamnestic questionnaire, and underwent a physical examination, ultrasound thyroid scan, serum determinations of fT3, fT4 and TSH, fine needle aspiration biopsy. The sample was subdivided into one group exposed to a determined quantity of radiation (detected by counter), one group exposed to an undetectable quantity of radiation, and the non exposed control group.

**Results:**

The prevalence of thyroid nodules <1 cm in diameter, defined as incidentalomas, in the exposed group with detected doses, was 11.28% in males and 9.68% in females, while in the exposed group with undetectable dose the prevalence was 10.39% in males and 16.67% in females. In the non exposed group the prevalence of incidentalomas was 9.34% in males and 13.20% in females. These prevalences were not statistically different when analysed by a multiple test comparison with the bootstrap method and stratification for sex.

Instead, the prevalence of thyroid nodules > 1 cm in diameter resulted statistically different in exposed and non exposed health staff: 18.68% in non exposed males vs exposed: 3.76% (determined dose) and 9.09% (undetectable dose) in males, and 20.30% in non exposed females versus 3.23% (detected dose) and 9.52% (undetectable dose) in exposed females.

There was a higher proportion of healthy staff in the exposed group than in the non exposed: (80.45% vs 68.68% in males; 80.65% vs 57.87% in females).

**Conclusion:**

In our study, occupational exposure to radiation combined with mild iodine deficiency did not increase the risk of developing thyroid nodules. The statistically significant higher prevalence of thyroid nodules in the non exposed group could be explained by the high percentage (22%) of people with a familial history of, and hence a greater predisposition to, thyroid disease.

The endemic condition of mild iodine deficiency, demonstrated in other studies, played a major role in determining the thyroid pathology in our study groups.

## Background

Thyroid nodules and thyroid cancer occur more frequently in people exposed to radiation for therapeutic purposes, and to nuclear fallout. Furthermore, it is known that a moderate degree of iodine deficiency may be responsible for an increased prevalence of thyroid nodules [8,13,14,20] and it is also suspected that radiation exposure could induce changes in thyroid autoimmunity [5,21]. However, well-designed long term studies are needed to accurately evaluate the complex association between low-dose environmental exposure to radiation and thyroid autoimmunity [5,21].

Recent studies in the rat have shown that thyroid cells are influenced both by irradiation and iodine intake, underlining the importance of the cell proliferation caused by disorders in iodine intake, either deficient or in excess [3].

There is a general consensus in the fact that there is a high prevalence of thyroid cancer and thyroid nodule formation in workers who are occupationally exposed to radiation [1,2,4,22], although some authors have shown confidence intervals of the odds ratio that are not so consistent with the risk of radiation [6,7].

The iodine intake of people resident in Bari, S. Italy, is mildly deficient [12], which could be presumed to cause a higher prevalence of thyroid pathology, as has in fact been demonstrated [9,10]. Therefore, in conditions of prolonged occupational exposure we could expect an increased prevalence [16]. This hypothesis is also linked to the observation that prolonged exposure to radiation from a young age increases the risk of thyroid disease [15,17,18].

This study was conducted with the aim of investigating the occurrence of thyroid nodules in hospital workers exposed to radiation, living in an area of mild iodine deficiency, and comparing the results with a representative group of non exposed subjects working in the same hospital.

## Methods

The study was conducted in the Azienda Ospedaliera Policlinico, Bari, and was designed as a cross-sectional study to evaluate the prevalence of thyroid nodules in staff occupationally exposed to radiation, and hence registered in the Radioprotection Service list. This group consists of 304 people, classified as belonging to the maximum risk category for radiation exposure, according to the Italian radioprotection law. They are periodically submitted to a complete physical examination by the physicians employed by the service, and the level of exposure is controlled by the physicists. We took into account the first examination of each exposed subject made in the year 2002.

A control group was set up, sending a letter inviting 986 non radiation-exposed staff, stratified for age, sex and assigned job unit, to participate in the investigation. The invitation was first sent in January 2002 and then repeated four times, until May 2003.

All the subjects who agreed to participate (n = 419; 42.5%) underwent a physical examination and a questionnaire was filled in collecting the following information: age, sex, job seniority, length of occupationally exposed period, cumulative annual radiation dose, presence of nodules confirmed by ultrasound scan, serum levels of fT3, fT4, TSH, familiarity, presence of other causes of radiation exposure (such as neck X-ray or CT scan, or radiotherapy).

To correctly define the kind of thyroid disease, a diagnostic protocol was established, including physical examination, ultrasound scan of the gland. Patients with thyroid nodules >1 cm in diameter were submitted to ultrasound examination and guided fine-needle aspiration (FNA).

Serum fT3, fT4, TSH levels were measured directly by RIA, only in the subjects with ultrasonographic thyroid alterations and CT was performed in the subjects with nodular lesions.

### Statistical analysis

Data were collected on Excel for Windows spreadsheets, and analysed with SAS software version 8.2 for PC.

Data were summarized as number and percentage for the qualitative variables. To evaluate the relation between categorical variables the chi-square test, adjusted for sex (χ^2^_MH_), was performed.

Multiple comparison was performed to evaluate the differences in percentage of thyroid conditions among the different groups of exposure. The Cochrane-Armitage test was used, with a bootstrap p-value adjustment [22]. Quantitative variables, were summarised as median and interquartile range (IQR), and non parametric tests were performed, because the Gaussian distribution could not be accepted (Wilks test: p < 0.01).

## Results

The percentage of the potential control group that agreed to participate in this study was 42.5% (419/986), but full information was received only from 38.8% of subjects (383/986). People were actively invited to contribute their data with repeated calls, but after one year (three repeat calls) the study was stopped.

Table 1 shows the main characteristics of the people recruited in the control group and exposed groups. Between the two groups there was a statistically significant difference for sex: the male percentage was 73.68% in exposed vs 47.52% in non exposed; the female percentage in exposed subjects was 26.32% vs 52.48% in non exposed (χ^2 ^= 46.08; p < 0.0001). No statistically significant difference was detected for age. In the exposed no information was available about familiarity for thyroid disease, whereas a history of thyroid disease was present in 22.19% (85/383) of the control group. At the time of the study, 477 people had no thyroid nodules, 77.96% (237/304) in the exposed group and 62.66% (240/383) among the controls, while about 35 healthy people lacked a precise value for exposure.

**Table 1:** Distribution of subjects according to the main characteristics

	Exposed		Not exposed		Not Included	
	
Sex	n	%	n	%	n	%
Male	224	73,68	182	48,02	12	36,36
Female	80	26,32	197	51,98	21	63,64
**Total**	**304**	**100,00**	**379**	**100,00**	**33**	**100,00**
*Missing*	-	-	*4*	-	*2*	-
	Exposed		Not exposed		Not Included	
	
Age class	n	%	n	%	n	%

up to 40	77	25,67	84	25,53	10	29,41
41–50	157	52,33	155	47,11	13	38,24
more or equal to 51	66	22,00	90	27,36	11	32,35
**Total**	**300**	**100,00**	**329**	**100,00**	**34**	**100,00**
*Missing*	*4*	-	*54*	-	*1*	-
	Exposed		Not exposed		Not Included	
	
Work status	n	%	n	%	n	%

Graduate health workers	133	47,00	37	9,79	6	18,18
Not graduate health workers	137	48,41	170	44,97	23	69,70
Administrative	-	0,00	65	17,20	-	0,00
Technical	-	0,00	29	7,67	-	0,00
Assistant	13	4,59	77	20,37	4	12,12
**Total**	**283**	**100,00**	**378**	**100,00**	**33**	**100,00**
*Missing*	*21*	-	*5*	-	*2*	-

Nodular pathology, single or multiple, was detected in 18 subjects in the exposed group (5.92%; 18/304) and 76 in the non exposed group (19.85%; 76/383).

Regarding nodules with a diameter less than 1 cm (defined as incidentalomas) we found an equal prevalence in the exposed and non exposed groups (11.51%, 35/304 vs 11.49%, 44/383).

The exposed group was divided into two subgroups according to the doses detected by counter: 0 μSv and more than 0 μSv. Among the group with complete information about thyroid pathology and level of exposure we restricted the analysis to 283 exposed people, for whom we had the precise level of radiation doses for the last 8 years.

Therefore, we compared the prevalence of nodules in the three groups: one group directly exposed to radiation (G1), one group working in the radiation risk environment but without any detected radiation (G2), one group of non exposed subjects (G3).

The thyroid conditions were slightly modified, classifying people in 4 classes: healthy, affected by thyroiditis, nodular pathology (taking together single and multiple nodules), incidentalomas. (Table 2).

**Table 2:** Distribution of people according to sex, group of exposure and thyroid condition

	MALE					
	
Thyroid condition	Exposed group with dose detected (G1)		Exposed group with doses = 0 (G2)		Not Exposed (G3)	
	
	N	%	N	%	N	%
Incidentalomas	15	11,3	8	10,4	17	9,3
Nodules (single or multiple)	5	3,8	7	9,1	34	18,7
Thyroiditis	6	4,5	3	3,9	6	3,3
Healthy	107	80,5	59	76,6	125	68,7
Total	133	100,0	77	100,0	182	100,0
	FEMALE					
	
Thyroid condition	Exposed group with dose detected (G1)		Exposed group with doses = 0 (G2)		Not Exposed (G3)	
	
	N	%	N	%	N	%

Incidentalomas	3	9,7%	7	16,7%	26	13,2
Nodules (single or multiple)	1	3,2%	4	9,5%	40	20,3
Thyroiditis	2	6,5%	3	7,1%	17	8,6
Healthy	25	80,6%	28	66,7%	114	57,9
Total	31	100,0%	42	100,0%	197	100,0

A statistically significant association was found between levels of exposure and thyroid condition adjusted for sex (χ^2^_MH _= 24.89, p = 0.0004).

A normal thyroid was more frequent in groups G1 and G2 than G3 (G1 vs G3: p = 0.009; G1+G2 vs G3: p = 0.0167).

There was a statistically significant difference in the occurrence of nodules, in particular when we compared G1 or G2, separately or together, with G3 (G1 vs G3: p < 0.0001; G2 vs G3: p = 0.0025; G1+G2 vs G3: p < 0.0001). In fact, the G3 group showed a higher prevalence of nodules compared with the other two groups.

There was a slightly higher prevalence of incidentalomas in exposed males, at both levels of exposure (G1: 11.28%15/133; G2: 10.39 8/77; G3: 9.34% 17/182). Instead, in women there was a higher frequency of both incidentalomas and nodules in the non exposed group. These differences did not result statistically significant.

Neither the frequency of thyroiditis was significantly different among G1, G2 and G3.

Only one woman, in the non exposed group with nodular pathology, was positive for papillary carcinoma at FNA. Therefore, in our sample there was a nodule malignancy rate of 2.94%(in G3).

Data on the history of exposure were available since 1995. For G1 the median period of radiation exposure was 8 years in all the pathologic groups and the non pathologic group. The median cumulative radiation dose in people with incidentalomas resulted lower (143 μSv IQR 69–1100 μSv) compared with that of people with other conditions. No significant difference in radiation exposure was found among the groups (table 3).

**Table 3:** Cumulative radiation doses (μSv) in the exposed group differentiated by thyroid condition

	N	Min	1° quartile	Median	3° quartile	Max
Incidentalomas	18	21.00	69.00	143.50	1100.00	5671.00
Nodules (single or multiple)	6	136.00	144.00	751.50	1248.00	4525.00
Thyroiditis	8	5.00	230.50	444.50	2683.00	12111.00
Healthy	132	0.00	54.50	175.00	1137.50	24635.00

## Discussion

A number of authors have underlined the role of radiation as a risk factor for the development of thyroid cancer, nodules or thyroiditis [2], particularly among X-ray workers and other health staff exposed to radiation in laboratories [5].

On the contrary, other authors have demonstrated that it is difficult to point to radiation as the cause of nodules or other non-malignant pathologies [13].

The Chernobyl episode gave rise to many epidemiological studies, analysing either the radiation risk for the general population or for workers. While the effect of radiation on the thyroid tissue of children is well proven [22], there is different evidence about the effects on the workers employed in the cleanup [21].

Little is known about the joint effect of iodine deficiency and radiation exposure on the risk of thyroid nodules: some authors have suggested that elimination of any iodine deficiency may be important in reducing the effects of radiation exposure on the thyroid [17].

Other authors have reported that mild iodine deficiency, or endemic iodine deficiency, can create a situation in which it is not possible to demonstrate the effect of radiation, because iodine intake is most effective as a risk factor [8,14].

Our results are not in agreement with this evidence, since we found a significantly different frequency of single nodules between the exposed groups and the control group, with a higher prevalence in the non exposed group. These results could be due to the higher percentage (22%) of people with familial thyroid disease and hence a greater predisposition to thyroid disease in the latter group.

Moreover, a selection bias could have entered the study, with a greater participation, especially in the control group, of people that were aware of the problem and decided to take advantage of the free medical tests.

We must also consider the fact that Bari and its surroundings are considered an area with mild iodine deficiency [12]. Our sample, both exposed and non exposed, is composed of people resident for more than 10 years in the Bari area, therefore environmental factors are very relevant in the development of thyroid pathologies.

Sex was found to be a significant factor for development of thyroid pathologies, both in the exposed and non exposed groups. Probably the well known characteristics of patients at risk of thyroid disease (females aged 40–60) have a stronger effect than radiation exposure in an occupational context where appropriate radioprotection measures are adopted.

Other authors have explained the lack of association of thyroid nodules and occupational radiation exposure by selection bias of the radiology service staff among healthy personnel, or by other selection filtering factors [22].

It is proven that long radiation exposure from a young age is a high risk factor for the development of thyroid cancer and nodules. In our sample, the exposure was well documented for the past 8 years, according to the Italian law, but the age of workers at the beginning of exposure, before the law came into effect, is not well documented.

In a recent meta-analysis by Tan and Gharib [19] the risk for malignancy in incidentalomas ranged between 0.45% and 13%. Contradictory attitudes have been proposed for the management of non palpable thyroid nodules. We agree with the suggestions that a systematic FNAB performed in all nodule <1 cm is not advisable, because only solid hypoechoic feature is a useful criterion to predict malignancy indipendently from the diameter of the nodule [11]. Considering that in our cohort of patients with incidentalomas, we didn't find these ecographic characteristics, we suppose the probably benign nature of most such lesions and we decided to kept them under periodical 'observation' without performing the FNAB. The real meaning of the high prevalence of incidentalomas in the exposed group requires a longer observation of these subjects and an enlargement of the casistic. The analysis of cumulative doses documented for each person did not result statistically significant. This can be considered a further element demonstrating the strict control of health conditions among exposed subjects, making it difficult to assess the risk of occupational radiation exposure because the effect of radioprotection measures can bias the conclusion.

## Conclusion

Our cross-sectional study shows evidence that occupational radiation exposure does not increase the risk of developing thyroid nodules. The statistically significant prevalence of thyroid disease in the non exposed group in our study could be explained by the high percentage (22%) of subjects with a familial history of thyroid disease and hence a greater predisposition to such diseases.

A repeat cross-sectional study in the future could help to control any modifications of the prevalence of nodules and of incidentalomas in the exposed groups. In this way we could detect the onset of disease, and also take into account any job shifts to the non exposed group.

## Competing interests

The author(s) declare that they have no competing interests.

## Authors' contributions

Trerotoli P., Serio G.: design of the study, data analysis and writing of the manuscript

Ciampolillo A.: patients examinations, interpretation of results of statistical analyses, writing of the manuscript

Marinelli G.: data collection and patients examination (exposed only)

Giorgino R.: revision of results and manuscript.

## Pre-publication history

The pre-publication history for this paper can be accessed here:


